# Comparing the sociodemographic characteristics of participants and non-participants in the population-based Tromsø Study

**DOI:** 10.1186/s12889-023-15928-w

**Published:** 2023-05-29

**Authors:** Chi Quynh Vo, Per-Jostein Samuelsen, Hilde Leikny Sommerseth, Torbjørn Wisløff, Tom Wilsgaard, Anne Elise Eggen

**Affiliations:** 1 Department of Community Medicine, Faculty of Health Sciences, UiT The Arctic University of Norway, N-9037 Tromsø, Norway; 2 Regional Medicines Information and Pharmacovigilance Centre (RELIS), University Hospital of North Norway, Tromsø, Norway; 3 The Norwegian Historical Data Centre, Department of Archaeology, History, Religious Studies and Theology, UiT The Arctic University of Norway, Tromsø, Norway; 4 Health Services Research Unit, Akershus University Hospital, Lørenskog, Norway

**Keywords:** Epidemiological studies, Sociodemographic characteristics, Survey, Area socioeconomic status

## Abstract

**Background:**

Differences in the sociodemographic characteristics of participants and non-participants in population-based studies may introduce bias and reduce the generalizability of research findings. This study aimed to compare the sociodemographic characteristics of participants and non-participants of the seventh survey of the Tromsø Study (Tromsø7, 2015–16), a population-based health survey.

**Methods:**

A total of 32,591 individuals were invited to Tromsø7. We compared the sociodemographic characteristics of participants and non-participants by linking the Tromsø7 invitation file to Statistics Norway, and explored the association between these characteristics and participation using logistic regression. Furthermore, we created a geographical socioeconomic status (area SES) index (low-SES, medium-SES, and high-SES area) based on individual educational level, individual income, total household income, and residential ownership status. We then mapped the relationship between area SES and participation in Tromsø7.

**Results:**

Men, people aged 40–49 and 80–89 years, those who were unmarried, widowed, separated/divorced, born outside of Norway, had lower education, had lower income, were residential renters, and lived in a low-SES area had a lower probability of participation in Tromsø7.

**Conclusions:**

Sociodemographic differences in participation must be considered to avoid biased estimates in research based on population-based studies, especially when the relationship between SES and health is being explored. Particular attention should be paid to the recruitment of groups with lower SES to population-based studies.

**Supplementary Information:**

The online version contains supplementary material available at 10.1186/s12889-023-15928-w.

## Background

Population-based studies are important, as they are often used as a source of data on determinants of health and as a source of information on people’s health status [1]. As such, these surveys should adequately reflect the target population for the relevant indicators. A problem with population-based studies is that participation is voluntary, thus people can choose not to participate. Non-participation can reduce the precision of estimates, and more seriously may introduce selection bias if both the exposure and the outcome under investigation affect the probability of participation, and may reduce the generalizability of the results [2]. The presence of selection bias cannot usually be inferred from the study data alone; participation studies are therefore necessary to identify any underrepresented subgroups [3]. Knowledge of the characteristics of non-participants may help to improve recruitment procedures and representativeness, leading to more accurate assumptions and conclusions in population-based studies, i.e., estimations of prevalence and incidence, and associations between exposures and outcomes.

Sociodemographic characteristics refer to a combination of social and demographic factors [4], including socioeconomic status (SES), which is often measured by an individual’s educational attainment, occupation, and income [5]. Individuals with low SES have been reported to have poorer health status and to be less likely to participate in health surveys compared with individuals with high SES [6–10]. Men, people who are unmarried, and those with low education or low income are also less likely to participate, according to previous studies [10–13]. The association between participation and age [14–16] or belonging to an ethnic minority [11, 17] is inconsistent in the literature.

National registers with high-quality individual-level data can be useful in providing information on non-participants, which can be compared with information on participants. The present study used register data to compare the sociodemographic characteristics of participants and non-participants of the seventh survey of the Tromsø Study (Tromsø7).

## Methods

### Study population

The Tromsø Study is an ongoing population-based health survey. It currently consists of seven surveys (Tromsø1-7) conducted between 1974 and 2016 in the municipality of Tromsø, Northern Norway. The study population consists of complete birth cohorts and random samples [18, 19]. Tromsø7 was carried out between 2015 and 2016, inviting all inhabitants aged 40 years and above in the municipality of Tromsø to participate. A total of 32,591 eligible individuals were invited and 65% participated in Tromsø7 [20].

### Linkage to statistics Norway

Information on sociodemographic characteristics recorded in Statistics Norway (SSB), which covers the entire Norwegian population, was linked with data from the Tromsø7 invitation file, which covered all 32,591 invited individuals, using the unique 11-digit personal identification number assigned to each resident of Norway at birth or immigration. SSB performed the linkage and all personal identification numbers were deleted.

### Sociodemographic characteristics of participants and non-participants

All sociodemographic characteristics of participants and non-participants of Tromsø7 were taken from the SSB, including age (10-years age intervals), sex, and marital status (married, unmarried, widow(er), and divorced/separated). The category “divorced/separated” included the subgroups separated (*n* = 517), separated partnership (*n* = 4), and divorced partner (*n* = 25). The category “married” included registered partnerships (*n* = 20). Data was also collected on country of birth, which was categorized into four broad groups: Norway, Western countries (Western Europe, North America, and Oceania), Eastern Europe (including Russia), and other countries (Asia, Africa, and South America). Individuals born in Norway were further categorized into three regions of birth: Tromsø, Northern Norway (Finnmark, Troms, and Nordland), and South Norway (counties south of Nordland). Finally, information was extracted on the highest completed educational level (primary education, upper secondary education, college/university < 4 years; and college/university ≥ 4 years), income (defined as individual income and total household income and categorized as in the Tromsø Study questionnaire: ≤ 250,000 Norwegian kroner (NOK) to ≥ 750,000 NOK), and residential ownership status (owner or renter).

### Statistical analyses

Descriptive characteristics were presented as number (percent). Sex-specific binary logistic regression analyses were used to estimate odds ratios (ORs) and corresponding 95% confidence intervals (CIs) of participation in unadjusted and age-adjusted models. The variable area SES was adjusted for individual-level socioeconomic status.

Individual-level SES was calculated based on educational level, individual income, total household income, and residential ownership status. For each of these four variables, a Z-score was calculated and then summarized to give an individual-level SES score. We also created a geographical SES index, based on 36 geographical subdivisions of the municipality of Tromsø defined in a local Public Health report [21]. These geographical subdivisions are based on the basic geographical and statistical units of the municipality of Tromsø, in order to establish small, stable geographical units that give a flexible basis for the presentation of regional statistics [22]. The geographical SES index was calculated as the average individual-level SES score in each of the geographical subdivisions, resulting in a continuous variable ranging from -1.73 to 1.24, and then categorized as low-SES area, medium-SES area, or high-SES area, based on tertiles using the command xtile in the statistical program Stata. Participation in Tromsø7 within each of the 36 geographical subdivisions was also divided into tertiles: low (59.3%), medium (66.7%), and high (68.5%), and the spatial distribution of SES areas and participation in the 36 geographical subdivisions was graphed using choropleth maps.

Analyses were performed in Stata 16.0 (StataCorp, College Station, TX, USA). Choropleth maps were created in Python 3 (using mainly the *pandas*, *geopandas*, and *plotly express* packages). A GeoJSON file was collected from the Norwegian Mapping Authority [23], while a base map from OpenStreetMap [24] was used.

## Results

**Fig. 1:**
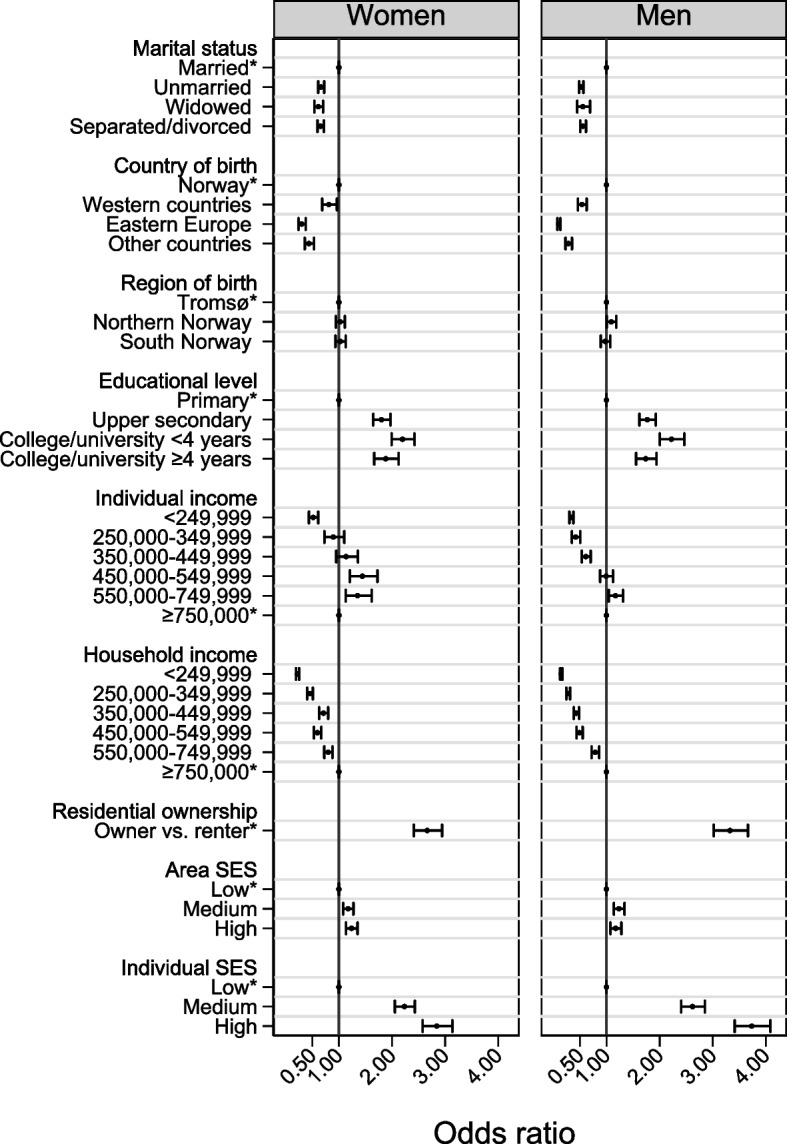
Age-adjusted odds ratios for participation by sex, Tromsø7 (2015–2016). *Reference group. **Additionally adjusted for individual-level socioeconomic status

A total of 32,591 individuals were invited to Tromsø7, of which 11,508 (35%) did not participate. The mean age of participants and non-participants was 57.3 years and 57.6 years, respectively. The median individual and total household income for participants were 431,799 NOK (IQR: 8680—585,830 NOK) and 725,354 NOK (IQR: 489,059—943,548 NOK), respectively. The corresponding figures for non-participants were 244,083 NOK (IQR: 0 – 524,675 NOK) and 546,086 NOK (IQR: 321,302 – 831,602 NOK). The sociodemographic distribution of participants differed from that of non-participants (Table 1). In both women and men, those who were unmarried, widowed, separated/divorced, born outside of Norway, had lower education, had lower income, were residential renters, and lived in a low-SES area had a lower probability of participation (Fig. 1 and Supplementary Table 1).

**Table 1:** Distribution of sociodemographic characteristics among participants and non-participants by sex, Tromsø7 (2015–2016)

	**Women** **(*n* = 16,537)**		**Men** **(*n* = 16,054)**	
	**Participants *n* = 11,073 (%)**	**Non-participants** ***n* = 5464 (%)**	**Participants *n* = 10,010 (%)**	**Non-participants *n* = 6044 (%)**
**Age, years**				
40–49	3377 (30.5)	1816 (33.3)	3055 (30.5)	2509 (41.4)
50–59	3245 (29.3)	1289 (23.6)	2790 (27.9)	1537 (25.4)
60–69	2677 (24.2)	909 (16.6)	2502 (25.0)	1041 (17.2)
70–79	1361 (12.3)	640 (11.7)	1315 (13.1)	582 (9.6)
80–99	413 (3.7)	810 (14.8)	348 (3.5)	375 (6.2)
**Marital status**				
Married	5768 (52.1)	2096 (38.4)	6023 (60.2)	2634 (43.6)
Unmarried	1429 (22.8)	1429 (26.1)	2491 (24.9)	2259 (37.3)
Widowed	850 (7.7)	899 (16.5)	207 (2.0)	193 (3.2)
Separated/divorced	1930 (17.4)	1040 (19.0)	1289 (12.9)	958 (15.9)
**Country of birth**^a^				
Norway	10 328 (93.3)	4848 (88.7)	9464 (94.5)	5048 (83.5)
Western countries	403 (3.6)	215 (3.9)	354 (3.5)	362 (6.0)
Eastern Europe	138 (1.2)	197 (3.6)	63 (0.6)	366 (6.1)
Other countries	204 (1.8)	204 (3.7)	129 (1.3)	268 (4.4)
**Region of birth**^b^				
Tromsø	4084 (39.5)	1817 (37.5)	3966 (41.9)	2201 (43.6)
Northern Norway^c^	3674 (35.6)	1719 (35.4)	3125 (33.0)	1556 (30.8)
South Norway^d^	2570 (24.9)	1312 (27.1)	2373 (25.1)	1291 (25.6)
**Educational level**				
Primary	1875 (17.0)	1655 (30.9)	1612 (16.2)	1516 (25.9)
Upper secondary	4071 (36.9)	1784 (33.2)	4428 (44.9)	2406 (41.1)
College/university < 4 years	3589 (32.6)	1293 (24.1)	2299 (23.1)	1030 (17.6)
College/university ≥ 4 years	1486 (13.5)	632 (11.8)	1576 (15.8)	900 (15.4)
**Individual income (NOK)**^e^				
< 249,999	4474 (40.4)	3151 (58.2)	3214 (32.1)	2583 (43.0)
250,000–349,999	660 (6.0)	288 (5.3)	309 (3.1)	289 (4.8)
350,000–449,999	1642 (14.8)	596 (11.0)	844 (8.4)	613 (10.2)
450,000–549,999	1978 (17.9)	589 (10.9)	1597 (16.0)	772 (12.8)
550,000–749,999	1746 (15.8)	550 (10.2)	2263 (22.6)	926 (15.6)
$\ge$ 750,000	568 (5.1)	239 (4.4)	1775 (17.8)	820 (13.6)
**Total household income (NOK)**^e^				
< 249,999	543 (4.9)	958 (17.7)	319 (3.2)	797 (13.3)
250,000–349,999	1040 (9.4)	756 (14.0)	559 (5.6)	728 (12.1)
350,000–449,999	1219 (11.0)	576 (10.6)	774 (7.7)	680 (11.3)
450,000–549,999	1161 (10.5)	611 (11.3)	947 (9.5)	667 (11.1)
550,000–749,999	2268 (20.5)	896 (16.6)	2333 (23.3)	1084 (18.1)
$\ge$ 750,000	4839 (43.7)	1616 (29.8)	5071 (50.7)	2048 (34.1)
**Residential ownership status**				
Owner	10,208 (92.2)	4269 (80.6)	9245 (92.4)	4635 (77.5)
Renter	860 (7.8)	1025 (19.4)	761 (7.6)	1349 (22.5)
**Area SES**				
Low	3526 (31.8)	2128 (38.9)	3142 (31.4)	2447 (40.5)
Medium	4028 (36.4)	1891 (34.6)	3731 (37.3)	1986 (32.8)
High	3519 (31.8)	1445 (26.5)	3137 (31.3)	1611 (26.7)
**Individual-level SES**				
Low	3289 (29.9)	2597 (50.5)	2249 (22.6)	2490 (43.3)
Medium	4358 (39.5)	1509 (29.3)	3669 (36.9)	1699 (29.5)
High	3372 (30.6)	1042 (20.2)	4039 (40.5)	1567 (27.2)

Percentage calculated to equal 100% in column

*NOK* Norwegian kroner, *SES* Socioeconomic status, *EUR* Euro, *USD* United States dollar

^a^Western countries (Western Europe, North America, and Oceania), Eastern Europe (including Russia), and Others (Asia, Africa, and Southern America)

^b^Among individuals born in Norway

^c^Northern Norway: County of Troms, Nordland, and Finnmark, excluding Tromsø

^d^South Norway: Counties south of Nordland County

^e^100,000 NOK = 10,480 EUR/11,526 USD

Men were less likely to participate than women (age-adjusted OR 0.79, 95% Cl 0.75 – 0.82, analysis not shown). Invitees aged 80–99 years were less likely to participate (women: OR 0.27, 95% Cl 0.24 – 0.31; men: OR 0.76, 95% Cl 0.65 – 0.89) compared to the youngest age group (40–49 years) and other age groups. However, the youngest age group was less likely to participate than those aged 50–79 years in both sexes. The odds of participation were highest among those with an educational level of college/university < 4 years, for both women (OR 2.20, 95% Cl 1.99 – 2.42) and men (OR 2.22, 95% Cl 2.00 – 2.47).

Participation decreased with decreasing individual and total household income for men. Among women, those with medium individual income (450,000–549,999 NOK) were more likely to participate than those with the highest individual income, while women with lowest individual income were less likely to participate. Lastly, individuals living in medium- and high-SES areas had higher odds to participate than those living in low-SES areas, after adjustment for individual-level SES. However, the estimated effect of area SES was not very strong (women: OR 1.24, 95% Cl 1.13 – 1.35; men: OR 1.17, 95% Cl 1.08 – 1.28). Individual-level SES showed a stronger effect, and those with high individual-level SES were around three times more likely to participate than those with low individual-level SES, in both sexes.

**Fig. 2:**
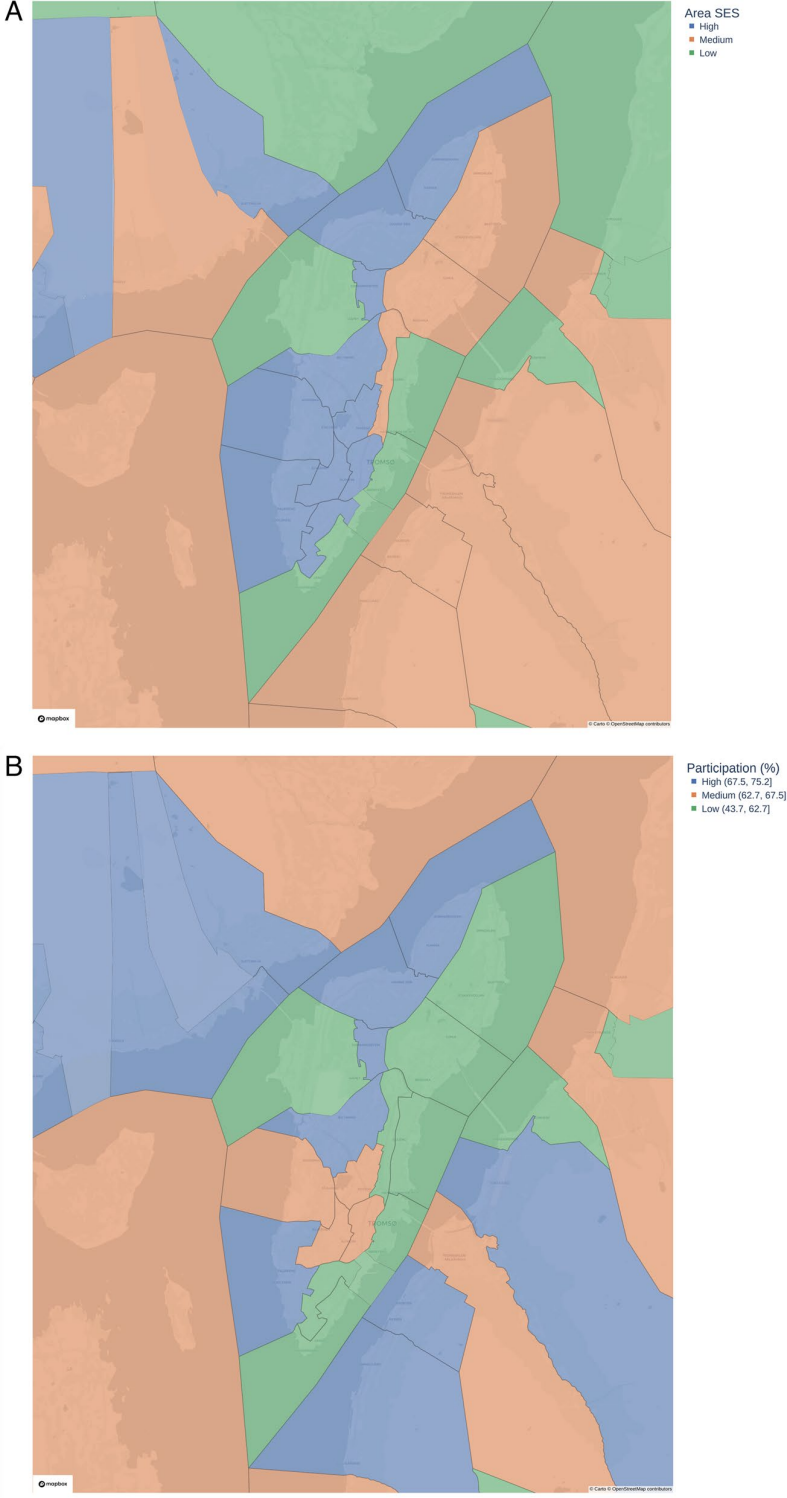
Choropleth maps of socioeconomic status (SES) areas (**A**) and participation (%) in the Tromsø Study (**B**) in 36 subdivisions of the municipality Tromsø, Tromsø7 (2015–2016). Maps: Kartverket (CC-BY 4.0), Carto/OpenStreetMap©(CC BY-SA 2.0), MapBox©. SES area based on the average individual-level SES score in each geographical subdivision [21]. SES: socioeconomic status

Generally, individuals living in high-SES areas, located on the West side of the city, had higher participation. None of the low-SES areas had high participation, but not all high SES areas had high participation, and there was more variation in participation in medium-SES areas (Fig. 2).

## Discussion

This study showed that men, people aged 49–49 and 80–89 years, those who were unmarried, widowed, separated/divorced, born outside of Norway, had lower education, had lower income, were residential renters, and lived in a low-SES area had a lower probability of participation in Tromsø7.

In accordance with results from Norwegian [9, 25, 26], Finnish [27, 28], and Dutch [29] studies, our study found that men were less likely to participate than women. In a previous Finnish study, women were found to engage more frequently in health behavior and to seek health-related information more often than men [30]. The tendency of men to have lower interest in participating in population-based studies has also been shown previously [31], and previous surveys of the Tromsø Study have had lower participation among men [18, 19]. In an attempt to increase participation among men in the age group 40–49 years, they were specifically targeted during the planning of Tromsø7 [20].

In the literature, evidence regarding study participation and age is much less consistent. We found that people aged 40–49 and 80–99 years were less likely to participate, whereas some studies have found that age does not affect participation [16], others found that individuals (40–49 years old) were more likely to participate [15], and still others found higher participation among older (> 60 years) individuals [14, 32]. Less participation among the oldest age group could be associated with poorer health among the very old [27, 28]; however, findings from another study suggested that older people’s health conditions do not affect survey participation [33]. Different explanations for participation in health surveys have been explored earlier [31, 34, 35]. Older persons (≥ 65 years) think that it is a civic duty to participate in population-based research, while lower participation among younger individuals may be due to a lack of time and a perception that their health is good [31, 34].

It has been suggested that marriage may encourage positive health behaviors, which over time cumulate and facilitate desirable health outcomes [36]. We observed that people with marital statuses other than married were less likely to participate than married individuals of both sexes. This is in accordance with other population-based studies [16, 25, 37]. Previous studies have highlighted the increased health and survival among married individuals compared to unmarried individuals [38, 39], which seems to be the case for men in particular [39, 40]. A possible explanation was proposed in a qualitative study on participants and non-participants of community health screening, which found that the decision to participate in screening is often made by a partner [41]. Sala et al. [33] reported that, among couples, if one partner took part in a health survey the other was more likely to respond as well.

According to several studies, participants born in the country where a survey is conducted are more likely to participate than those born outside of the country [9, 11, 12, 15]. Even though the municipality of Tromsø is currently the 12th most populous in Norway, it has relatively few immigrants (16%, year 2021) compared to other populous municipalities in the country [42, 43]. Furthermore, the Tromsø Study questionnaires are in Norwegian, and to participate in the Tromsø Study, individuals had to master the Norwegian language. In an Australian study, speaking the same language at home as was used in the questionnaire was associated with higher odds of participation [15]. This indicates that language difficulties hinder participation.

In our study, increased educational level, total household income, and being a residential owner were all socioeconomic factors associated with an increased probability of participation. Prior literature has also reported that participation was more likely among individuals with high educational level, income [7–11, 44], and among residential owners [14, 15, 37]. Bopp et al. [37] suggested that residential owners are more likely to participate because they move less frequently, and are therefore easier to track. Education is considered an important social determinant of health, as it helps to promote and sustain healthy lifestyles and positive health choices [45]. Nadelsen et al. [46] found that as years of college increased, trust in science also increased. Furthermore, the authors suggested that people with more education are more likely to have a deeper understanding of science and the work of scientists, and are thus more likely to be engaged in critical examinations of scientific issues. For instance, UiT The Arctic University of Norway and The University Hospital of North Norway are among the largest public workplaces in the municipality of Tromsø [47], and their employees belong to occupational groups with a higher educational level. As research is as a part of their work tasks, they have an deeper understanding of science and their willingness to participate might be higher than that observed in other workplaces. In addition, different employers in Tromsø were asked to give their employees time off from work to participate in Tromsø7 [48]. Indeed, a Norwegian qualitative study showed that reasons for not participating in a population-based study included difficulty in taking a day off from work and loss of salary during participation [34]. This might apply especially to individuals in low-income groups, as they are more financially vulnerable than those with higher income. Furthermore, in this qualitative study, an informant suggested that if people were to get paid by their employer to participate in health research, more people might participate [34]. Some have suggested providing modest financial compensation for lost work time and travel expenses as a token of appreciation to increase participation rates [28, 34]. Olsen et al. [49] found that a scratch lottery ticket incentive increased participation among individuals with lower education, and this might apply to low-income groups as well. However, these approaches are expensive, especially for a population-based study whose target group is the entire general population.

Individuals with low SES do not only participate less compared to individuals with high SES, but their participation decreases over time, according to a follow-up study of a randomized controlled trial [50]. Indeed, participation in health surveys decreases over time in all educational levels, though the decline seems fastest for those with low education [44].

It has been suggested that participation may not depend only on individual characteristics, but also on geographical features [51]. The SES of the surrounding area has been reported to be associated with lower participation in cohort studies [14, 16, 51]. This is consistent with our findings, which showed that those living in high-SES areas were more likely to participate than those living in low-SES areas. Sala et al. [33] found that participation among women was associated with their socioeconomic background and the wealth of their residential area. Bender et al. [52] hypothesize that residents of deprived neighborhoods may be lack of social support to participate in health check and they may also be less trusting of public health authorities.

The literature is generally consistent in showing that those living in economically disadvantaged areas have poorer health [53, 54]. For instance, the prevalence of diabetes mellitus has been found to be lower for those living in areas with medium and high SES for both women and men, compared to those living in areas with low SES [55]. Those who volunteer to participate in health surveys are often more likely to have favorable exposures and health profiles compared to those who do not [14, 56, 57]. Sociodemographic differences in participation can lead to bias in the population-level estimates and in the associations with health status and health behaviors. For instance, low educational level and low income are both positively associated with unhealthy dietary habits [58]. If low educational level is also associated with non-participation, as we have shown, and unhealthy dietary habits are associated with the probability of participation, any resultant associations will be biased (selection bias). A false conclusion might thus be drawn about the health status of the population. Furthermore, non-participation can lead to an underestimation of the prevalence of health indicators and harmful health behaviors [29], as well as reduced precision of estimates.

Efforts should be made to recruit subgroups that we have shown to be underrepresented in our study. For example, our findings show non-participation by area SES. This can help the Tromsø Study and other population-based studies when planning recruitment for future surveys. However, sending extra reminders has shown little impact on the sociodemographic distribution among participants, so other methods to increase the participation of underrepresented groups should be explored [59].

### Strength and limitations

The main strength of this study is the linkage of information on sociodemographic characteristics from the Tromsø Study to that from the SSB, a national register. Another strength is the use of a large population-based study with reasonably high participation. Our study provides an overview of the representativeness of the Tromsø Study regarding a variety of sociodemographic characteristics. A potential limitation of this study is that we have categorized continuous variables; as there are no perfect cut-off points for variables like income, information might be lost in categorization. Errors in the collection and processing of the income data are unavoidable, even for administrative data. Income information for employed individuals was based on complete registration from employers and other administrative data. An extensive work from SSB has been carried out to minimize errors, and we consider errors to be relatively insignificant. Whereas information from self-employed individuals in Norway is self-reported, but they are required to provide accurate taxable income, which is carefully controlled by the tax authorities.

In conclusion, sociodemographic differences in participation must be considered to avoid biased estimates in research based on population-based studies, especially when the relationship between SES and health is being explored. Particular attention should be paid to the recruitment of groups with lower SES to population-based studies.

## Supplementary Information

**Additional file 1: Supplementary Table 1.** Odds ratios for participation by sex, Tromsø7 (2015-2016).

## Data Availability

The data that support the findings of this study are available from the Statistics Norway (SSB), but restrictions apply to the availability of these data, which were used under license for the current study, and so are not publicly available.

## Abbreviations

CL: Confidents intervals
DPIA: Data Protection Impact Assessment
IQR: Interquartile range
NSD: The Norwegian Data Protection Authority
OR: Odds ratio
REK North: The Regional Committee for Medical and Health Research Ethics North
SES: Socioeconomic status
SSB: Statistics Norway
