# Supplementary material for: Comparing the sociodemographic characteristics of participants and non-participants in the population-based Tromsø Study
Source: BMC Public Health. 2023 May 29;23:994. doi: 10.1186/s12889-023-15928-w (PMC10226228; doi:10.1186/s12889-023-15928-w)
Supplement: Supplementary file 1 — Additional file 1: Supplementary Table 1. Odds ratios for participation by sex, Tromsø7 (2015-2016). [file 12889_2023_15928_MOESM1_ESM.docx]

Supplementary Table 1. Odds ratios for participation by sex, Tromsø7 (2015-2016).

| **Women Men Women Men** | | | | |
| --- | --- | --- | --- | --- |
|  | **Crude OR**  **(95% Cl)** | **Crude OR**  **(95% Cl)** | **Adjusted OR (95% Cl)^a^** | **Adjusted OR (95% Cl)^b^** |
| **Age, years** |  |  |  |  |
| 40-49 | Ref. | Ref. | - | - |
| 50-59 | 1.35 (1.24 – 1.48) | 1.49 (1.37 – 1.62) | - | - |
| 60-69 | 1.58 (1.44 – 1.74) | 1.97 (1.80 – 2.16) | - | - |
| 70-79 | 1.14 (1.02 – 1.28) | 1.86 (1.66 – 2.07) | - | - |
| 80-99 | 0.27 (0.24 – 0.31) | 0.76 (0.65 – 0.89) | - | - |
| **Marital status** |  |  |  |  |
| Married | Ref. | Ref. | Ref. | Ref. |
| Unmarried | 0.64 (0.59 – 0.70) | 0.48 (0.45 – 0.52) | 0.66 (0.61 – 0.72) | 0.52 (0.49 – 0.57) |
| Widowed | 0.34 (0.31 – 0.38) | 0.47 (0.38 – 0.57) | 0.61 (0.54 – 0.70) | 0.56 (0.45 – 0.69) |
| Separated/divorced | 0.67 (0.62 – 0.74) | 0.59 (0.53 – 0.65) | 0.65 (0.60 – 0.72) | 0.56 (0.51 – 0.61) |
| **Country of birth** |  |  |  |  |
| Norway | Ref. | Ref. | Ref. | Ref. |
| Western countries | 0.88 (0.74 – 1.04) | 0.52 (0.45 – 0.61) | 0.81 (0.68 – 0.96) | 0.54 (0.46 – 0.63) |
| Eastern Europe | 0.26 (0.26 – 0.41) | 0.09 (0.07 – 0.12) | 0.30 (0.24 – 0.38) | 0.10 (0.08 – 0.13) |
| Other countries | 0.39 (0.39 – 0.57) | 0.26 (0.21 – 0.32) | 0.44 (0.36 – 0.53) | 0.28 (0.23 – 0.35) |
| **Region of birth** |  |  |  |  |
| Tromsø | Ref. | Ref. | Ref. | Ref. |
| Northern Norway | 0.95 (0.88 – 1.03) | 1.11 (1.03 – 1.21) | 1.02 (0.94 – 1.11) | 1.09 (1.00 – 1.18) |
| South Norway | 0.87 (0.80 – 0.95) | 1.02 (0.94 – 1.11) | 1.03 (0.93 – 1.13) | 0.98 (0.89 – 1.07) |
| **Educational level** |  |  |  |  |
| Primary | Ref. | Ref. | Ref. | Ref. |
| Upper secondary | 2.01 (1.85 – 2.20) | 1.75 (1.61 – 1.91) | 1.80 (1.65 – 1.97) | 1.77 (1.62 – 1.93) |
| College/university <4 years | 2.45 (2.24 – 2.69) | 2.10 (1.90 – 2.32) | 2.20 (1.99 – 2.42) | 2.22 (2.00 – 2.47) |
| College/university ≥4 years | 2.08 (1.85 – 2.33) | 1.65 (1.48 – 1.83) | 1.88 (1.67 – 2.12) | 1.74 (1.56 – 1.94) |
| **Individual income (NOK)** |  |  |  |  |
| <249,999 | 0.60 (0.51 – 0.70) | 0.57 (0.52 – 0.63) | 0.52 (0.44 – 0.61) | 0.34 (0.30 – 0.38) |
| 250,000-349,999 | 0.96 (0.79 – 1.18) | 0.49 (0.41 – 0.59) | 0.90 (0.73 – 1.10) | 0.42 (0.35 – 0.51) |
| 350,000-449,999 | 1.16 (0.97 – 1.38) | 0.64 (0.56 – 0.73) | 1.14 (0.95 – 1.36) | 0.61 (0.53 – 0.70) |
| 450,000-549,999 | 1.41 (1.18 – 1.69) | 0.96 (0.85 – 1.08) | 1.44 (1.21 – 1.71) | 0.99 (0.88 – 1.12) |
| 550,000-749,999 | 1.34 (1.12 – 1.60) | 1.13 (1.01 – 1.26) | 1.35 (1.13 – 1.62) | 1.17 (1.04 – 1.31) |
| ≥750,000 | Ref. | Ref. | Ref. | Ref. |
| **Total household income (NOK)** |  |  |  |  |
| <249,999 | 0.19 (0.17 – 0.21) | 0.16 (0.14 – 0.19) | 0.22 (0.19 – 0.25) | 0.15 (0.13 – 0.17) |
| 250,000-349,999 | 0.46 (0.41 – 0.51) | 0.31 (0.27 – 0.35) | 0.45 (0.40 – 0.51) | 0.28 (0.25 – 0.31) |
| 350,000-449,999 | 0.71 (0.63 – 0.79) | 0.46 (0.41 – 0.52) | 0.71 (0.63 – 0.80) | 0.43 (0.38 – 0.49) |
| 450,000-549,999 | 0.63 (0.57 – 0.71) | 0.57 (0.51 – 0.64) | 0.59 (0.53 – 0.67) | 0.49 (0.44 – 0.55) |
| 550,000-749,999 | 0.85 (0.77 – 0.93) | 0.87 (0.80 – 0.95) | 0.80 (0.72 – 0.88) | 0.79 (0.72 – 0.86) |
| ≥750,000 | Ref. | Ref. | Ref. | Ref. |
| **Residential ownership status** |  |  |  |  |
| Owner | 2.84 (2.58 – 3.14) | 3.53 (3.21 – 3.89) | 2.66 (2.41 – 2.94) | 3.32 (3.02 – 3.66) |
| Renter | Ref. | Ref. | Ref. | Ref. |
| **Area SES^2^** |  |  |  |  |
| Low | Ref. | Ref. | Ref. | Ref. |
| Medium | 1.29 (1.19 – 1.39) | 1.46 (1.36 – 1.58) | 1.17 (1.08 – 1.27) | 1.23 (1.14 – 1.34) |
| High | 1.47 (1.35 – 1.59) | 1.52 (1.40 – 1.64) | 1.24 (1.13 – 1.35) | 1.17 (1.08 – 1.28) |
| **Individual-level SES** |  |  |  |  |
| Low | Ref. | Ref. | Ref. | Ref. |
| Medium | 2.28 (2.11 – 2.46) | 2.39 (2.20 – 2.59) | 2.23 (2.05 – 2.43) | 2.62 (2.41 – 2.85) |
| High | 2.56 (2.34 – 2.79) | 2.85 (2.63 – 3.10) | 2.84 (2.58 – 3.14) | 3.73 (3.41 – 4.08) |

^a^Adjusted for age.

^b^Additionally adjusted for individual-level SES.

OR: odds ratio, CI: confidence interval, NOK: Norwegian kroner, SES: socioeconomic status.
